# Assessment of the cardiovascular safety of saxagliptin in patients with type 2 diabetes mellitus: pooled analysis of 20 clinical trials

**DOI:** 10.1186/1475-2840-13-33

**Published:** 2014-02-04

**Authors:** Nayyar Iqbal, Artist Parker, Robert Frederich, Mark Donovan, Boaz Hirshberg

**Affiliations:** 1 Bristol-Myers Squibb, Route 206 & Providence Line Rd, Princeton, NJ 08543, USA; 2 AstraZeneca, 1800 Concord Pike, Wilmington, DE 19850, USA

**Keywords:** Dipeptidyl peptidase-4 inhibitor, Major adverse cardiovascular events, Saxagliptin, Type 2 diabetes mellitus

## Abstract

**Background:**

It is important to establish the cardiovascular (CV) safety profile of novel antidiabetic drugs.

**Methods:**

Pooled analyses were performed of 20 randomized controlled studies (N = 9156) of saxagliptin as monotherapy or add-on therapy in patients with type 2 diabetes mellitus (T2DM) as well as a subset of 11 saxagliptin + metformin studies. Adjudicated major adverse CV events (MACE; CV death, myocardial infarction [MI], and stroke) and investigator-reported heart failure were assessed, and incidence rates (IRs; events/100 patient-years) and IR ratios (IRRs; saxagliptin/control) were calculated (Mantel-Haenszel method).

**Results:**

In pooled datasets, the IR point estimates for MACE and individual components of CV death, MI, and stroke favored saxagliptin, but the 95% CI included 1. IRR (95% CI) for MACE in the 20-study pool was 0.74 (0.45, 1.25). The Cox proportional hazard ratio (95% CI) was 0.75 (0.46, 1.21), suggesting no increased risk of MACE in the 20-study pool. In the 11-study saxagliptin + metformin pool, the IRR for MACE was 0.93 (0.44, 1.99). In the 20-study pool, the IRR for heart failure was 0.55 (0.27, 1.12).

**Conclusions:**

Analysis of pooled data from 20 clinical trials in patients with T2DM suggests that saxagliptin is not associated with an increased CV risk.

## Introduction

Cardiovascular (CV) disease is the leading cause of mortality and morbidity in patients with type 2 diabetes mellitus (T2DM) [1]. In the United States, the prevalence of self-reported CV disease in people with T2DM is estimated to be >30% [2], and CV events account for almost 70% of diabetes-related deaths in individuals aged ≥65 years [1].

Although epidemiologic studies suggest that hyperglycemia is associated with adverse CV events [3-5], the effects of intensive glycemic control on CV outcomes in interventional studies are not clear [6-8]. Moreover, in some studies and with some antihyperglycemic drugs, a tendency toward an increased risk for CV events has been reported [7,9,10]. However, follow-up of prominent clinical trials in type 1 [11] and T2DM [12] suggest that intensive glycemic control may reduce CV events over the long term.

Because of the uncertainty surrounding glycemic control and CV events and the association of increased CV events with some antihyperglycemic drugs, in 2008 the US Food and Drug Administration recommended that CV safety be assessed as a component of the clinical development program of new antihyperglycemic drugs [13].

Saxagliptin is a dipeptidyl peptidase-4 (DPP-4) inhibitor approved as an adjunct to diet and exercise to improve glycemic control in adults with T2DM [14]. DPP-4 inhibitors are oral antihyperglycemic agents that inhibit the inactivation of the incretin hormones, glucagon-like peptide-1 (GLP-1) and glucose-dependent insulinotropic peptide, resulting in increased glucose-dependent insulin secretion and suppression of glucagon secretion [15]. Observational evidence suggests that GLP-1 may have protective effects on the CV system, independent of glucose control [16]. However, DPP-4 is increased in patients with T2DM [17,18] and elevated circulating DPP-4 is associated with subclinical left ventricular dysfunction in these patients [18]. Therefore, it is of interest to assess the CV safety of DPP-4 inhibitors.

In randomized, controlled, clinical trials, saxagliptin was effective and well tolerated over 24 weeks in improving glycemic control when used as monotherapy [19,20] and as add-on therapy to metformin [21], glyburide [22], or a thiazolidinedione [23] in patients with T2DM. The advantages of DPP-4 inhibitors are their tolerability, a low rate of hypoglycemia, and weight neutrality [24].

Results from large outcome trials of saxagliptin in patients with prior CV disease or multiple CV risk factors (SAVOR) [25] and alogliptin in patients after acute coronary syndrome (EXAMINE) have recently been published [26] and have shown that saxagliptin and alogliptin do not increase or decrease major adverse CV events (MACE). In contrast to those trials in patients with T2DM and high CV risk, the current analysis evaluated MACE and its individual component events of CV death, myocardial infarction (MI) and stroke, as well as heart failure, with saxagliptin in the general population of patients with T2DM that participated in the saxagliptin clinical development program. The present analysis expands on a previous assessment of the CV safety of saxagliptin [27] and analyzes MACE in 20 phase 2 and 3 trials of saxagliptin versus placebo or active comparator.

## Materials and methods

### Study design

This post hoc analysis (N = 9156) used pooled data from 20 randomized phase 2b and 3b controlled clinical trials of saxagliptin. These trials were placebo-controlled or active-comparator studies of saxagliptin (2.5, 5, or 10 mg/d in most studies; 20, 40, or 100 mg/d in 1 phase 2b study) as monotherapy or add-on therapy to metformin, a sulfonylurea, a thiazolidinedione, or insulin ± metformin for up to 206 weeks (including long-term extension studies) in patients with T2DM (Table 1). Data from the SAVOR study in patients with prior CV disease or multiple CV risk factors were not included in this analysis. In some studies, rescue medication (metformin, pioglitazone, or titrated insulin) was given during the study if patients met prespecified glycemic criteria. In long-term extension studies of monotherapy, patients in the placebo arm received blinded metformin 500 mg. Detailed methodology and primary findings for these studies have been published (Table 1). Patients were followed until completion of the study or premature discontinuation from the study. The studies were performed in accordance with the Declaration of Helsinki and all patients provided written informed consent. The protocols were approved by a local ethics committee.

**Table 1:** Studies included in the 20-study pool

**Study**	**Study design**	N*	**Mean baseline HbA**_**1c**_**, %**	**Treatment**	**Reference**
NCT00950599	Phase 2, randomized, 6-wk (high-dose cohort) or 12-wk (main cohort) dose ranging study in treatment-naïve patients	423	7.5–8.0	SAXA 2.5, 5, 10, 20, 40, or 100 mg/d vs PBO	Rosenstock et al, 2008 [28]
NCT00575588^†^	Phase 3, randomized, 52 wk + 52-wk LTE	858	7.7	SAXA 5 mg/d + MET vs glipizide 5–20 mg/d + MET	Göke et al, 2010; 2013 [29,30]
NCT00666458^†^	Phase 3, randomized, 18 wk	801	7.7	SAXA 5 mg/d + MET vs SITA 100 mg/d + MET	Scheen et al, 2010 [31]
NCT00698932	Phase 3, randomized, 24 wk in treatment-naïve Asian patients. Rescue medication: metformin	568	8.1–8.2	SAXA 5 mg/d vs PBO	Pan et al, 2012 [32]
NCT00661362^†^	Phase 3, randomized, 24 wk in Asian patients	570	7.9	SAXA 5 mg/d + MET vs PBO + MET	Yang et al, 2011 [33]
NCT00614939	Phase 3, randomized, 12 wk + 40-wk LTE in patients with renal impairment	170	8.1–8.5	SAXA 2.5 mg/d vs PBO (± other OADs or INS)	Nowicki et al, 2011 [34,35]
NCT00918879	Phase 3, randomized, 24 wk in treatment-naïve Indian patients. Rescue medication: metformin	213	8.3	SAXA 5 mg/d vs PBO	Prasanna Kumar et al, 2014 [36]
NCT00121641	Phase 3, randomized, 24 wk + 42-mo LTE in treatment-naïve patients. Rescue medication: metformin; placebo arm: metformin 500 mg/d during LTE	401^‡^	7.8–8.0	SAXA 2.5, 5, or 10 mg/d vs PBO	Rosenstock et al, 2009; 2013 [20,37]
NCT00295633	Phase 3, randomized, 24 wk + 52-wk LTE. Rescue medication: metformin	565	8.2–8.4	SAXA 2.5 or 5 mg/d + TZD vs PBO + TZD	Hollander et al, 2009; 2011 [23,38]
NCT00121667^†^	Phase 3, randomized, 24 wk + up to 42-mo LTE. Rescue medication: pioglitazone	743	8.0	SAXA 2.5, 5, or 10 mg/d + MET vs PBO + MET	DeFronzo et al, 2009; Rosenstock et al, 2013 [21,37]
NCT00316082	Phase 3, randomized, 24 wk + 52-wk LTE in treatment-naïve patients. Rescue medication: metformin; placebo arm: metformin 500 mg/d during LTE	365	7.8–8.0	SAXA 2.5 mg QAM ± titration to 5 mg,^§^ 5 mg QAM, or 5 mg QPM vs PBO	Frederich et al, 2012 [19]
NCT00327015^†^	Phase 3, randomized, 24 wk + 52-wk LTE in treatment-naïve patients. Rescue medication: pioglitazone	1306	9.4–9.6	SAXA 5 or 10 mg/d + MET vs SAXA 10 mg/d + PBO or MET + PBO	Jadzinsky et al, 2009; Pfützner et al, 2011 [39,40]
NCT00313313	Phase 3, randomized, 24 wk + 52-wk LTE. Rescue medication: metformin	768	8.4–8.5	SAXA 2.5 or 5 mg/d + GLY vs PBO + GLY uptitrated to 15 mg/d^‡^	Chacra et al, 2009; 2011 [22,41]
NCT00374907	Phase 3, randomized, 12 wk + 104-wk LTE in treatment-naïve patients. Rescue medication: metformin; placebo arm: metformin 500 mg/d during LTE	36	6.6–6.9	SAXA 5 mg/d vs PBO	Henry et al, 2011 [42]
NCT00757588^†^	Phase 3, randomized, 24 wk + 28-wk LTE. Rescue medication: titrated insulin	455	8.6–8.7	SAXA 5 mg/d + INS ± MET vs PBO + INS ± MET	Barnett et al, 2012 [43]
NCT00683657^†^	Phase 3, randomized, 4 wk	93	8.1	SAXA 5 mg/d + MET XR vs PBO + MET XR	Stenlof et al, 2010 [44]
NCT00885378^†^	Phase 3, randomized, 12 wk	160	7.9–8.0	SAXA (2.5 mg twice daily) + MET vs PBO MET	White et al, 2014 [45]
NCT00918138^†^	Phase 3, randomized, 4 wk	93	8.4–8.6	SAXA 5 mg/d + MET XR 1500 mg vs MET XR uptitration to 2000 mg^¶^	Neutel et al, 2013 [46]
NCT01006590^†^	Phase 3/4, randomized, 24 wk	286	7.7–7.8	SAXA 5 mg/d + MET 1500 mg vs MET uptitration to 2000 or 2500 mg^§^	Hermans et al, 2012 [47]
NCT00960076^†^	Phase 3, randomized, 18 wk	282	8.3–8.4	SAXA 5 mg/d + MET XR vs MET XR uptitration to 1000 mg^§^	Fonseca et al, 2012 [48]

GLY = glyburide; HbA_1c_ = glycated hemoglobin; INS = insulin; LTE = long-term extension; MET = metformin; OAD = oral antidiabetic drug; PBO = placebo; QAM = once daily in the morning; QPM = once daily in the evening; SAXA = saxagliptin; SITA = sitagliptin; TZD = thiazolidinedione; XR = extended release.

*Number of randomized and treated patients.

^†^Included in the subset analysis of studies of saxagliptin as add-on therapy to metformin.

^‡^Main cohort only.

^§^Dose increase if hyperglycemia criteria were met, up to maximum indicated dose.

^¶^Dose increased to indicated maximum.

### Analyses

Adverse events (AEs) and serious AEs (SAEs) were reported by study investigators using standard reporting procedures. AEs were coded using the Medical Dictionary for Regulatory Activities, version 15.0 (MedDRA). AEs occurring up to 1 day following the last treatment day or up to the last visit day in the short-term plus long-term period (where applicable), whichever was later, were included. SAEs occurring up to 30 days following the last treatment day or up to the last visit day in the short-term plus long-term period, whichever was later, were included.

Major adverse CV events, defined as CV death, MI, stroke, and cardiac ischemic events reported by investigators were systematically identified using a list of MedDRA preferred term (PT) diagnoses. All identified potential CV events subsequently went through treatment-blinded adjudication by independent reviewers at the Duke Clinical Research Institute (DCRI; Durham, NC; 8 studies) or the Montreal Heart Institute (MHI; Montreal, QC, Canada; 12 studies).

Briefly, for the studies retrospectively reviewed by DCRI, cases included all deaths, MI, and stroke events as well as all events coded by any of the 148 MedDRA PTs representing possible ischemic events. Methods for full CV event identification have been previously published [27]. For the 12 studies prospectively adjudicated by MHI, the sponsor identified potential cases for adjudication based on AEs and SAEs with PTs that correlated with the following Standardized MedDRA Queries (SMQs) groupings, as defined by the current version of MedDRA: “ischemic heart disease” (adjudicated for possible MI) and “cerebrovascular disorders” (adjudicated for possible stroke). In addition, SAEs (only) with PTs that correlated with the SMQs of “cardiac arrhythmias” or “cardiac failure” were sent for adjudication to determine if the cardiac failure or cardiac arrhythmia was precipitated by MI. Additionally, any event that led to death was identified for adjudication [27]. Heart failure events were not adjudicated and were identified based on PTs from a narrow SMQ for “cardiac failure”.

Safety was analyzed in all treated patients, including those meeting rescue medication criteria. Analyses of CV events were performed using the pooled 20-study dataset and a separate pooled subset of 11 studies of saxagliptin add-on therapy to metformin (NCT00575588, NCT00666458, NCT00661362, NCT00121667, NCT00327015 [included saxagliptin + placebo and saxagliptin + metformin vs metformin + placebo], NCT00757588 [included saxagliptin + insulin ± metformin or insulin ± metformin], NCT00683657, NCT00885378, NCT00918138, NCT01006590, NCT00960076). In addition, subgroup analyses of MACE were performed for saxagliptin 2.5 mg versus control and saxagliptin 5 mg versus control in the 20-study pool. The saxagliptin 2.5-mg group included patients who received an initial dose of saxagliptin 2.5 mg once daily, except for those enrolled in the renal impairment study (NCT00614939). The saxagliptin 5-mg group included patients who received an initial dose of saxagliptin 5 mg once daily or 2.5 mg twice daily. Patients receiving doses of saxagliptin <2.5 mg/d or >5 mg/d were not included in the analyses by dose.

For MACE and individual CV component events, the number of patients with the event, the time up to an event or censoring (for patients without a MACE), the exposure-adjusted incidence rate (IR), and the incidence rate ratio (IRR), which provides a means to account for differences in study duration and mean follow-up time with saxagliptin and control, were calculated. To account for differences between studies in patients, event rates, and randomization ratios, the IR (number of patients with events per 100 patient-years) with 95% CI was calculated using the Mantel-Haenszel method, stratified by study. Exact 95% CI was calculated for the IRR, stratified by study. In addition, adjudicated MACE were analyzed using a Cox proportional hazards model.

## Results

### Patient demographics and clinical characteristics

In the 20-study pool, demographic and clinical characteristics were similar between the saxagliptin (n = 5701) and control (n = 3455) groups (Table 2). Most patients were white and <65 years of age, and 45% (control) to 49% (saxagliptin) had a duration of T2DM of ≤3 years. In the add-on to metformin study pool, demographic and clinical characteristics were also similar between the saxagliptin (n = 2981) and control (n = 2190) groups (Table 3). There was a higher proportion of patients with duration of T2DM of ≤1.5 years in the 20-study pool, compared with the 11-study saxagliptin add-on to metformin pool; otherwise, no notable differences were observed between the pooled populations. The total follow-up time for saxagliptin and control for the 20-study pool was 6051 and 2869 patient-years, respectively, with an average follow-up time of 1.06 years/patient and 0.83 years/patient, respectively. The proportion of patients that prematurely discontinued from the study varied based on the length of study. The rate of premature discontinuation was higher with saxagliptin versus control in 3 studies, higher with control versus saxagliptin in 9 studies, and similar between groups in the remaining studies.

**Table 2:** Patient demographics and clinical characteristics in the 20-study pool

**Characteristic, n (%)**	**All SAXA**	**Control**
	**(n = 5701)**	**(n = 3455)**
Age, y		
<65	4681 (82.1)	2766 (80.1)
≥65	1020 (17.9)	689 (19.9)
≥75	132 (2.3)	91 (2.6)
Women	2899 (50.9)	1696 (49.1)
Race		
White	3707 (65.0)	2034 (58.9)
Asian	1319 (23.1)	1001 (29.0)
Black	217 (3.8)	102 (3.0)
Other	458 (8.0)	318 (9.2)
BMI, kg/m^2^		
<30	2914 (51.1)	1888 (54.6)
≥30	2780 (48.8)	1564 (45.3)
Not reported	7 (0.1)	3 (<0.1)
Duration of T2DM, y		
≤1.5	2129 (37.3)	1076 (31.1)
≤3	2817 (49.4)	1558 (45.1)
>3– < 5	776 (13.6)	523 (15.1)
≥5	2107 (37.0)	1373 (39.7)
≥10	834 (14.6)	582 (16.8)
Not reported	1 (<0.1)	1 (<0.1)
Creatinine clearance, mL/min		
<30	37 (0.6)	41 (1.2)
30– < 50	90 (1.6)	68 (2.0)
50–80	973 (17.1)	658 (19.0)
>80	4598 (80.7)	2685 (77.7)
Not reported	3 (<0.1)	3 (<0.1)

BMI = body mass index; SAXA = saxagliptin; T2DM = type 2 diabetes mellitus.

**Table 3:** Patient demographics and clinical characteristics in the pool of saxagliptin add-on to metformin studies

**Characteristic, n (%)**	**All SAXA**	**Control**
	**(n = 2981)**	**(n = 2190)**
Age, y		
<65	2397 (80.4)	1733 (79.1)
≥65	584 (19.6)	457 (20.9)
≥75	86 (2.9)	62 (2.8)
Women	1533 (51.4)	1070 (48.9)
Race		
White	2031 (68.1)	1426 (65.1)
Asian	549 (18.4)	469 (21.4)
Black	109 (3.7)	58 (2.6)
Other	292 (9.8)	237 (10.8)
BMI, kg/m^2^		
<30	1438 (48.2)	1114 (50.9)
≥30	1541 (51.7)	1074 (49.0)
Not reported	2 (<0.1)	2 (<0.1)
Duration of T2DM, y		
≤1.5	828 (27.8)	504 (23.0)
≤3	1198 (40.2)	833 (38.0)
>3– < 5	475 (15.9)	386 (17.6)
≥5	1308 (43.9)	971 (44.3)
≥10	489 (16.4)	391 (17.9)
Creatinine clearance, mL/min		
30– < 50	28 (0.9)	14 (0.6)
50–80	493 (16.5)	405 (18.5)
>80	2457 (82.4)	1768 (80.7)
Not reported	3 (0.1)	3 (0.1)

BMI = body mass index; SAXA = saxagliptin; T2DM = type 2 diabetes mellitus.

### Cardiovascular events

**Figure 1:**
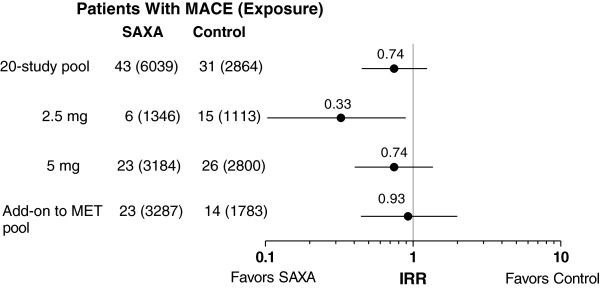
**Incidence rate ratios for saxagliptin vs control (point estimates and 95% CI) for MACE in the 20-study pool, the saxagliptin 2.5- and 5-mg subanalysis, and the add-on to metformin study pool.** Numbers in parentheses are total patient-years of exposure (the time up to an event or censoring). IRR = incidence rate ratio; MACE = major adverse cardiovascular events; MET = metformin; SAXA = saxagliptin.

In the 20-study pool, exposure time to the first MACE or censoring was 6039 patient-years in the saxagliptin group versus 2864 patient-years in the control group. A total of 43 patients who received saxagliptin had an adjudicated MACE versus 31 patients in the control group (Figure 1). The IRs per 100 patient-years (SE) were 0.85 (0.14) for saxagliptin and 1.12 (0.20) for control, with an IRR (95% CI) of 0.74 (0.45, 1.25). The Cox proportional hazard ratio (HR; 95% CI) was 0.75 (0.46, 1.21), suggesting no increased risk of MACE in the 20-study pool.

In the subgroup analyses of adjudicated MACE for saxagliptin 2.5 and 5 mg in the 20-study pool, the IR per 100 patient-years (SE) was 0.47 (0.19) for saxagliptin 2.5 mg versus 1.38 (0.36) for control and 0.73 (0.15) for saxagliptin 5 mg versus 0.97 (0.19) for control. The IRR (95% CI) for saxagliptin 2.5 and 5 mg were 0.33 (0.10, 0.89) and 0.74 (0.40, 1.36), respectively (Figure 1).

In the 11-study pool of saxagliptin add-on to metformin, the exposure time to a first MACE event or censoring was 3287 patient-years in the saxagliptin group versus 1783 patient-years in the control group. A total of 23 patients who received saxagliptin had an adjudicated MACE versus 14 patients in the control group (Figure 1). The IR per 100 patient-years (SE) was similar for saxagliptin (0.79 [0.17]) and control (0.85 [0.23]), yielding an IRR (95% CI) of 0.93 (0.44, 1.99).

**Figure 2:**
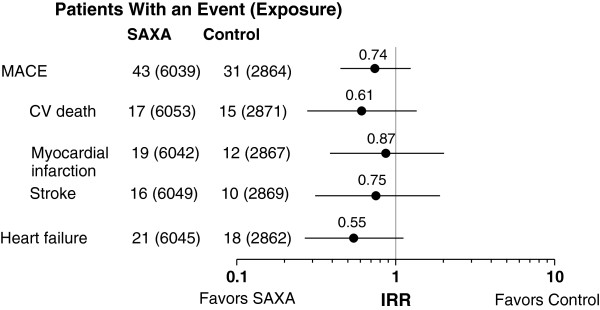
**Incidence rate ratios for saxagliptin vs control (point estimates and 95% CI) for CV death, myocardial infarction, stroke, and heart failure in the 20-study pool.** Numbers in parentheses are total patient-years of exposure (the time up to an event or censoring). CV = cardiovascular; IRR = incidence rate ratio; MACE = major adverse cardiovascular events; SAXA = saxagliptin.

**Figure 3:**
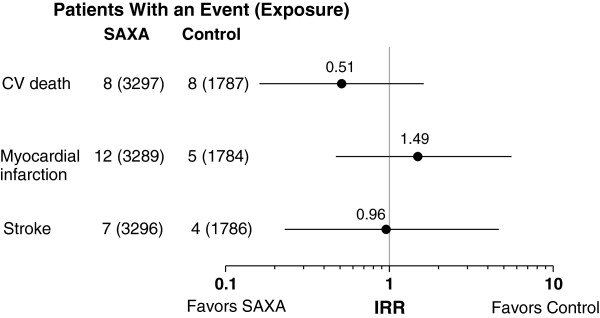
**Incidence rate ratios for saxagliptin vs control (point estimates and 95% CI) for CV death, myocardial infarction, and stroke in the add-on to metformin study pool.** Numbers in parentheses are total patient-years of exposure (the time up to an event or censoring). CV = cardiovascular; IRR = incidence rate ratio; SAXA = saxagliptin.

In the 20-study pool, the IR point estimates (SE) for the individual components of MACE were 0.34 (0.09) for saxagliptin versus 0.54 (0.14) for control for CV death; 0.40 (0.10) versus 0.45 (0.13), respectively, for MI; and 0.27 (0.07) versus 0.36 (0.11) for stroke (Figure 2). In the 11-study pool of saxagliptin add-on to metformin, IR estimates (SE) for saxagliptin versus control were 0.27 (0.10) versus 0.49 (0.18) for CV death, 0.44 (0.13) versus 0.31 (0.14) for MI, and 0.21 (0.08) versus 0.22 (0.11) for stroke. IRRs for these events ranged from 0.51 to 1.49 (Figure 3).

Heart failure was not defined as a component of MACE and was not adjudicated but was analyzed separately. For heart failure (20-study pool only), the IR (SE) was 0.34 (0.08) and 0.62 (0.15) for saxagliptin and control, respectively. IRRs for these individual events ranged between 0.55 and 0.87 (Figure 2).

## Discussion

In this pooled analysis of 9156 patients with T2DM from 20 phase 2 and 3 clinical trials, treatment with saxagliptin was not associated with an increased risk of CV events and heart failure compared with placebo or active comparator. These results expand on previous findings on the CV safety of saxagliptin reported in a meta-analysis of 8 phase 2 and 3 trials [27]. In that analysis, a total of 40 MACE events in 4607 patients were reported. The relative risk (95% CI) for saxagliptin versus comparator for a composite endpoint of adjudicated CV death, MI, and stroke was 0.43 (0.23, 0.80), which suggested possible CV protection with saxagliptin. The present analysis expanded on the previous study and included 9156 patients who experienced 74 MACE events. In this larger population, which should provide a more precise risk estimate, the relative risk (95% CI) for a composite endpoint of adjudicated CV death, MI, and stroke was 0.75 (0.46, 1.21), suggesting no increased risk of MACE in this 20-study pool. Incidence rates for CV events for saxagliptin were not different from those for placebo or comparator in most analyses, with the exception of the lower IR for MACE in the saxagliptin 2.5-mg group in the subanalysis of the 20-study pool. However, it should be noted that only 7 of the 20 studies included patients who had received the 2.5-mg saxagliptin dose.

The present findings are also consistent with previously published meta-analyses of CV events from clinical trial programs for other DPP-4 inhibitors, including vildagliptin, sitagliptin, linagliptin, and alogliptin. In a pooled analysis of 25 clinical trials, the relative risk (95% CI) for cardiocerebrovascular events for vildagliptin was 0.88 (0.37, 2.11) for 50 mg once daily and 0.84 (0.62, 1.14) for 50 mg twice daily [49]. In other meta-analyses, the IRR or HR (95% CI) for CV-related events versus comparators was 0.83 (0.53–1.30) for sitagliptin [50], 0.34 (0.16, 0.70) for linagliptin [51], and 0.64 (1-sided 97.5% CI, 0.0, 1.406) for alogliptin [52]. In addition, a meta-analysis of 70 trials of DPP-4 inhibitors enrolling 41,959 patients reported a reduction in MACE (n = 495 total events of CV death, nonfatal MI, and stroke and acute coronary syndromes and/or heart failure; odds ratio, 0.71 [95% CI, 0.59, 0.86]) [53]. Although these studies are not directly comparable because of different CV endpoints, study designs, adjudication procedures, patient populations and background medication, all supported the hypothesis that DPP-4 inhibitors do not increase CV risk and may possibly have CV benefits in patients with T2DM.

Results from the large outcome trial of saxagliptin in patients with prior CV disease or multiple CV risk factors (SAVOR) have recently been reported [25]. Results generally consistent with those were also reported from the alogliptin trial (EXAMINE) in patients after acute coronary syndrome [26]. SAVOR demonstrated neutrality on the composite primary endpoint of CV death, MI, or ischemic stroke (HR, 1.00 [95% CI, 0.89, 1.12]). The MACE results reported here in a much lower-risk population with an event rate approximately a third of that observed in SAVOR are consistent with SAVOR in demonstrating a safe profile of saxagliptin with respect to MACE events. The fact that SAVOR did not demonstrate superiority compared with placebo raises at least two alternative, though not mutually exclusive, interpretations: (1) evidence suggesting benefit from meta-analysis and preclinical evidence [16,54] was due to chance or (2) saxagliptin and likely other DPP-4 inhibitors are safe in all populations and trends to benefit occur only in the lower-risk general population studied in the phase 3 clinical development program. The latter hypothesis has been previously suggested based on the only positive interaction of subgroups in a patient level meta-analysis of UKPDS, ACCORD, ADVANCE, and VADT [55]. Owing to the marked difference in population characteristics (eg, age, CV history and risk factors, duration of diabetes, background diabetes and CV medications, proportion of patients with baseline glycated hemoglobin <7%) and population risk (3- to 6-fold higher event rate) between SAVOR and EXAMINE and the meta-analyses of phase 3 programs of saxagliptin and alogliptin, it is difficult to support or dismiss either interpretation for the lack of benefit observed in SAVOR and EXAMINE.

SAVOR also demonstrated neutrality on the broader composite endpoint of CV death, MI, stroke, or hospitalization for unstable angina, heart failure, or coronary revascularization (HR, 1.02 [95% CI, 0.94, 1.11]). One component of this broader endpoint, hospitalization for heart failure, did have an HR with 95% CI which did not include 1 (HR, 1.27 [95% CI, 1.07, 1.51]). As reported here, heart failure in the 20-study pool had an HR (95% CI) of 0.55 (0.27, 1.12). Again, differences in the patient population, background medications, and/or chance may be involved in the relative inconsistency of these results. Moreover, SAVOR was an event-driven trial in a highly defined population (prior CV disease or multiple CV risk factors), whereas the 20 clinical trials analyzed in this study had defined treatment periods ranging from 4 to 206 weeks and included diverse patient populations with T2DM (eg, patients who were treatment naïve, receiving varying background antihyperglycemic medications, or with renal impairment). The phase 3 data presented in this manuscript suggest that the observation of hospitalization for heart failure could not have been anticipated based on the phase 3 development program. It may be that further analysis of SAVOR results or the other prospective CV outcome trials with DPP-4 inhibitors [56,57] will give further clarity to the two issues raised here.

Certain limitations of this analysis should be recognized and considered when interpreting the results. To handle missing data as the result of premature discontinuation, analysis methods assumed similar event rates had the patient completed the study. However, patients treated with saxagliptin tended to be followed longer and had a lower rate of discontinuation compared with those who received control treatment. Results using this assumption should be interpreted with caution.

Both groups received a range of background medications, including metformin, sulfonylureas, and thiazolidinediones and the control group received both active medications and placebo. In several studies, a titration of background medication [22,41,46-48] or a titration of double-blind saxagliptin [19,29,30,39,40] was permitted. In addition, in the majority of studies, rescue medication was permitted [19-23,32,36,38-43]. These factors complicate interpretation of the findings.

The saxagliptin group was also heterogeneous and included patients treated with doses higher than the approved 2.5- and 5-mg once-daily doses. Further, the analyses of the 2.5- and the 5-mg doses used distinct study pools because not all studies included 2.5- and 5-mg arms, which precludes direct comparison of results for the 2 doses. It is important to recognize that the pooled patient population in these clinical trials was highly selected, which may have resulted in a lower event rate compared with that observed in clinical practice. Finally, there was relatively limited experience beyond 18 months.

## Conclusion

Pooled data from 20 clinical trials involving 9156 patients with T2DM suggest that saxagliptin is safe and not associated with an increased CV risk.

## Abbreviations

AEs: Adverse events; BMI: Body mass index; CI: Confidence intervals; CV: Cardiovascular; DCRI: Duke Clinical Research Institute; DPP-4: Dipeptidyl peptidase-4; GLP-1: Glucagon-like peptide-1; GLY: Glyburide; HbA1c: Glycated hemoglobin; INS: Insulin; IR: Incidence rate; IRR: Incidence rate ratio; LTE: Long-term extension; MACE: Major adverse cardiovascular events; MedDRA: Medical Dictionary for Regulatory Activities; MET: Metformin; MHI: Montreal Heart Institute; MI: Myocardial infarction; OAD: Oral antidiabetic drug; PBO: Placebo; QAM: Once daily in the morning; QPM: Once daily in the evening; SAEs: Serious adverse events; SAXA: Saxagliptin; SITA: Sitagliptin; SMQ: Standardized MedDRA Queries; T2DM: Type 2 diabetes mellitus; TZD: Thiazolidinedione; XR: Extended release.

## Competing interests

NI, RF, and MD are employees of Bristol-Myers Squibb. AP and BH are employees of AstraZeneca.

## Authors’ contributions

NI contributed to the conception and design of the study and the drafting and final approval of the manuscript. AP contributed to the conception and design of the study and the drafting, revision, and final approval of the manuscript. RF contributed to the conception and design of the study and the drafting, revision, and final approval of the manuscript. MD contributed to the conception and design of the study, analyzed the data, and revised and approved the final version of the manuscript. BH contributed to the conception and design of the study and the drafting, revision, and final approval of the manuscript.
